# A machine learning based authentication and intrusion detection scheme for IoT users anonymity preservation in fog environment

**DOI:** 10.1371/journal.pone.0323954

**Published:** 2025-06-16

**Authors:** Khondokar Oliullah, Md Whaiduzzaman, Md. Julkar Nayeen Mahi, Tony Jan, Alistair Barros

**Affiliations:** 1 Department of Information and Communication Technology, Comilla University, Cumilla, Bangladesh; 2 Institute of Information Technology, Jahangirnagar University, Dhaka, Bangladesh; 3 School of Information Systems, Queensland University of Technology, Brisbane, Queensland, Australia; 4 Design and Creative Technology Vertical, Torrens University, Brisbane, Queensland, Australia; National University of Sciences and Technology, UNITED KINGDOM OF GREAT BRITAIN AND NORTHERN IRELAND

## Abstract

Authentication is a critical challenge in fog computing security, especially as fog servers provide services to many IoT users. The conventional authentication process often requires disclosing sensitive personal information, such as usernames, emails, mobile numbers, and passwords that end users are reluctant to share with intermediary services (i.e., Fog servers). With the rapid growth of IoT networks, existing authentication methods often fail to balance low computational overhead with strong security, leaving systems vulnerable to various attacks, including unauthorized access and data interception. Additionally, traditional intrusion detection methods are not well-suited for the distinct characteristics of IoT devices, resulting in a low accuracy in applying existing anomaly detection methods. In this paper, we incorporate a two-step authentication process, starting with anonymous authentication using a secret ID with Elliptic Curve Cryptography (ECC), followed by an intrusion detection algorithm for users flagged as suspicious activity. The scheme allows users to register with a Cloud Service Provider (CSP) using encrypted credentials. The CSP responds with a secret number reserved in the Fog node for the IoT user. To access the services provided by the Fog Service Provider (FSP), IoT users must submit a secret ID. Furthermore, we introduce a staked ensemble learning approach for intrusion detection that achieves 99.86% accuracy, 99.89% precision, 99.96% recall, and a 99.91% F1-score in detecting anomalous instances, with a support count of 50,376. This approach is applied when users fail to provide a correct secret ID. Our proposed scheme utilizes several hash functions through symmetric encryption and decryption techniques to ensure secure end-to-end communication.

## Introduction

The rapid increase of Internet of Things (IoT) devices has revolutionized various aspects of daily life [1], enabling seamless connectivity and automation [2]. However, the widespread adoption of IoT technology has also introduced significant security and privacy challenges, particularly in fog computing environments [3]. Fog computing extends cloud capabilities to the network edge, providing computation, storage, and services closer to end users [4]. This paradigm shift enhances real-time data processing and reduces latency, making it ideal for IoT applications. However, it also raises concerns regarding secure authentication and intrusion detection, especially given the sensitive nature of the data involved.

One of the fundamental security concerns [5] in fog computing is ensuring the authentication of massive-scale IoT users [6]. Traditional authentication mechanisms often require the disclosure of personal information, such as usernames, emails, and passwords, which poses a significant privacy risk [7]. Users are increasingly wary of sharing sensitive information with service providers, including Cloud Service Providers (CSPs) and Fog Service Providers (FSPs), due to concerns about data breaches and misuse. Thus, there is a pressing need for authentication schemes that preserve user anonymity while ensuring secure access.

In addition to authentication, intrusion detection [8, 9] is a critical component of a comprehensive security strategy in fog computing. The diverse and resource-constrained nature of IoT devices complicates [10] the implementation of traditional intrusion detection systems (IDS). These systems often struggle with the heterogeneity of IoT environments and the wide variety of potential threats. Existing anomaly-based detection methods [11, 12], while promising, have not yet achieved the necessary consistent accuracy levels to be fully effective in these settings. Therefore, this has motivated the need for a lightweight yet robust authentication and intrusion detection model specifically tailored for IoT and fog infrastructures. Our proposed model addresses these challenges by integrating ECC-based encryption for secure, low-cost authentication, paired with a machine learning-driven intrusion detection system that continuously monitors and adapts to evolving threats. This combination ensures data protection and real-time threat identification, creating a secure and efficient framework that meets the unique requirements of IoT and fog computing environments.

This research introduces an anonymity-preserving authentication scheme [13, 14] tailored for IoT users in fog computing environments. The proposed scheme leverages encrypted credentials to protect user identities during registration and communication processes. Furthermore, we integrate a stacked ensemble learning-based intrusion detection mechanism capable of achieving high accuracy, even in the face of complex and evolving threats. The dual focus on privacy-preserving authentication and robust intrusion detection addresses critical gaps in existing security frameworks for fog computing.

The proposed anonymity-preserving scheme for IoT user authentication in Fog Computing significantly enhances security and efficiency by utilizing encryption and hashing techniques, requiring only a secret number or ID submission. Moreover, ensemble learning reduces the risk of overfitting and improves generalization by integrating multiple models. This robustness is vital in a diverse and dynamic environment like Fog-Cloud-IoT [15], where data characteristics can vary widely. Moreover, the Fog-Cloud-IoT environment generates complex [16], high-dimensional data. Ensemble learning, particularly through methods like stacking, is capable of managing such complexity by leveraging the strengths of different algorithms to capture various patterns in the data.

The main contributions are as follows:

- We design and implement an authentication mechanism to ensure IoT users’ anonymity in the fog computing context.
- We enable the authentication mechanism verifying with encrypted credentials during the registration and authentication phase for anonymity preservation in the Fog-IoT environment.
- We introduce a staked ensemble learning model for intrusion detection tailored to fog-IoT environments to detect high accuracy of anomalous identity.
- We evaluate the proposed scheme thoroughly using real-world IoT datasets and simulations.

The remainder of this paper is organized as follows: Section “Literature review” reviews related work in the areas of fog computing security, authentication schemes, and intrusion detection systems. Section “System model and methodology” details the proposed anonymity-preserving authentication framework and the machine learning model used for intrusion detection. Section “Experimental results and discussion” presents the experimental setup, evaluation metrics, and results, while Section “Discussion and future direction” describes the findings and future research directions. Finally, Section “Conclusions” concludes the paper.

## Literature review

The rapid proliferation of Internet of Things (IoT) devices has led to significant challenges [17, 18] in securing these systems, especially when combined with fog computing infrastructures. Fog computing, with its ability to process data closer to the data source, offers advantages over traditional cloud computing by reducing latency and improving response times. However, these benefits also introduce new security and privacy concerns [19], particularly regarding authentication and intrusion detection.

Authentication is a critical aspect of securing IoT systems, ensuring that only authorized devices and users can access network resources. Traditional authentication mechanisms, such as username-password schemes, are not well-suited for IoT environments due to the constrained nature of IoT devices and the need for minimal human intervention. Research by [20] introduced a lightweight authentication protocol for IoT devices in fog computing, focusing on reducing computational overhead.

Similarly, Adam *et al*. [21] proposed a secure authentication scheme leveraging elliptic curve cryptography (ECC) to ensure secure communications with low computational cost. Despite these advancements, a significant challenge remains: preserving user anonymity while maintaining robust security. Corthis *et al*. [22] addressed the security challenges in IoT-based cloud systems, particularly in healthcare, focusing on privacy, authentication, and secure data transmission. Traditional methods like homomorphic encryption [23] and elliptic curve cryptography are inadequate for healthcare IoT security needs.

The proposed solution uses fog computing with a hybrid model combining Elliptic Curve Cryptography (ECC) and Proxy Re-encryption (PR), enhanced by the Enhanced Salp Swarm Algorithm (ESSA). This approach improves IoT device verification, identification, and authentication, significantly reducing processing time and improving reliability. The model also offers efficient communication cost and memory usage, enhancing real-time data sharing security in healthcare IoT systems [24].

Maintaining anonymity in IoT systems is crucial to protect user privacy, particularly in applications involving sensitive data. Tanveer *et al*. [25] explored anonymous authentication techniques in smart grids, proposing a method that masks user identities using pseudonyms. However, extending these techniques to general IoT and fog computing environments requires careful consideration of scalability and resource constraints.

Anonymity-preserving authentication schemes, such as the one proposed by Limkar *et al*. [26], utilize cryptographic protocols to ensure that users can authenticate themselves without revealing their identities. Despite these efforts, balancing anonymity with authentication efficiency and scalability remains a key challenge. A number of recent studies [27, 28] presented some solutions for enhancing IoT device security, focusing on privacy and anonymity.

These introduced different architectures that used blockchain technology to manage IoT devices, such as a CCTV system in rental properties, and smart healthcare [29]. The system allowed either the owner or renter to access the IoT node feed based on smart contract conditions, ensuring no third-party involvement. The Key contributions included a new framework [30], a smart contract algorithm, and modifications to ring signatures for improved security. The proposed system showed better performance in key management compared to existing schemes and is designed to be scalable for other applications, such as healthcare monitoring [31, 32].

Another article [33] introduced a Lightweight Anonymous Mutual Authentication Scheme (LAMAS) for fog computing, leveraging elliptic curve cryptography to ensure secure and efficient mutual authentication between users and fog servers. It supports the seamless addition of new fog servers without re-registering users or requiring additional computations. Formal and informal security analyses validate the scheme’s robustness, and comparative evaluations show that LAMAS outperforms existing schemes in terms of lower computation cost and reduced storage requirements, making it a practical and secure solution for fog computing environments.

Intrusion detection systems (IDS) are essential for monitoring and identifying malicious activities within networks. The decentralized nature of fog computing provides an opportunity to deploy IDS closer to the edge, where data is generated, allowing for faster detection and response. Diro *et al*. (2021) [34] presented a distributed IDS framework for fog computing that leverages edge nodes for real-time threat detection.

However, traditional IDS techniques often struggle with the diverse and dynamic nature of IoT traffic. Machine learning-based IDS approaches have gained traction due to their ability to learn patterns and detect anomalies. Kalaivani *et al*. (2020) [35] proposed a deep learning-based IDS model that operates in a fog computing environment, demonstrating high accuracy in detecting a variety of attacks. Despite these successes, challenges such as high computational costs and the need for extensive training data persist. Moreover, ensuring the privacy of user data during the training and operation of ML-based IDS is a growing concern.

A number of latest studies [36, 37] proposed intrusion detection frameworks for IoT and fog computing environments, addressing the limitations of traditional IDS due to limited resources at fog nodes such as computational complexity [38], reliance on specific datasets, etc. These frameworks integrate different autoencoders for feature extraction, CatBoost for feature refinement, and an ensemble of transformer, CNN, and LSTM, BiLSTM [39] models for comprehensive analysis. These approaches are validated using the NSL-KDD, UNSW-NB15, BoT-IoT, and AWID datasets, achieving satisfactory accuracy in most cases except in the case of [40] in detecting cyber threats. The study demonstrates the effectiveness of combining edge preprocessing with cloud-based ensemble learning to secure real-world fog and IoT infrastructures against evolving cyber-attacks.

Moreover, the unique aspects of IoT-based security protocols have been published recently. Mahmood *et al*. [52] present a privacy-preserving protocol with a focus on resilience against data interception and forgery. Another study [53], by integrating digital twins and blockchain, offers a futuristic perspective for cross-domain IIoT security, while the study [54] targets secure, efficient data exchange within IoT-enabled smart grids. The first scheme could further enhance clarity by specifying threat types, while the other two studies could benefit from mentioning practical applications to reinforce real-world relevance. Together, these abstracts reflect the broad spectrum of innovative solutions currently emerging to address security in IoT-based environments, each contributing unique mechanisms and optimizations for enhancing security and efficiency.

While significant progress has been made in developing authentication mechanisms [6, 41, 42] and IDS for fog-IoT environments [43–45], several gaps remain. Most existing authentication protocols either compromise on user anonymity or are too resource-intensive for practical deployment in IoT scenarios [46]. Additionally, while machine learning-based IDS offer promising accuracy, they often require substantial computational resources, which may not be available in resource-constrained [47] fog nodes. Furthermore, there is a need for IDS solutions that can adapt to the evolving threat landscape without compromising user privacy.

The existing literature highlights the importance of developing secure, efficient, and privacy-preserving solutions for fog-IoT environments. This paper aims to address the identified gaps by proposing an anonymity-preserving authentication scheme combined with a machine learning-based intrusion detection system. The proposed solution seeks to offer robust security, preserve user anonymity, and operate efficiently within the resource constraints typical of fog computing nodes.

## System model and methodology

This section introduces the system model and methodology for an anonymity-preserving scheme designed for IoT user authentication and machine learning-based intrusion detection in fog computing environments. The proposed solution addresses the security challenges posed by resource constraints and the dynamic nature of IoT and fog architectures. It outlines the interactions between IoT devices, fog nodes, and the cloud server, describing the cryptographic protocols for secure and anonymous authentication and the machine learning techniques for effective intrusion detection. The aim is to provide a secure, reliable, and efficient framework that enhances real-time threat detection while preserving user anonymity.

### Architecture design of Fog-IoT environment

Each area will have its own fog node connected to a centralized cloud server. This setup ensures consistent latency and network usage for each fog node. However, the time and network usage required to upload and retrieve data from the centralized cloud server will increase.

**Fig 1:**
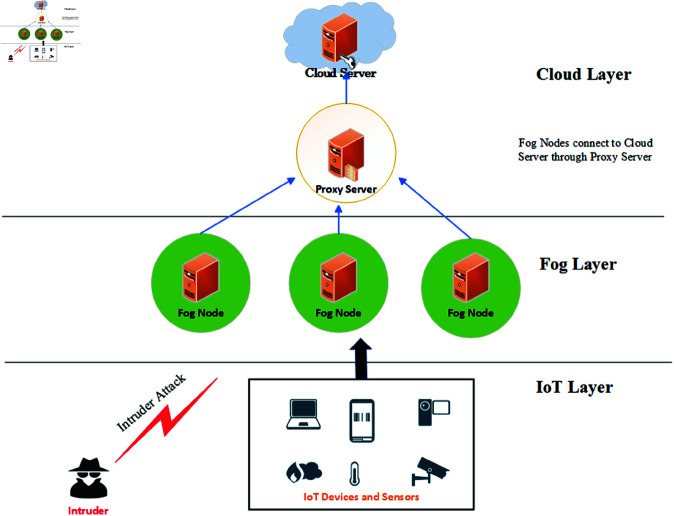
Fog-IoT architecture.

Fig 1 illustrates a comprehensive three-layer architecture for an IoT-fog-cloud computing system, designed to ensure secure communication and efficient data processing. At the bottom layer, the IoT Layer comprises various IoT devices and sensors, such as smart gadgets, cameras, and environmental monitors, which collect data from their surroundings. These devices are vulnerable to potential intruders, symbolized by an "Intruder" icon, representing the security threats that the system aims to detect and mitigate.

The middle layer, the Fog Layer, consists of multiple fog nodes strategically placed closer to the IoT devices to handle initial data processing and storage. This proximity reduces latency and enhances real-time data handling capabilities. A critical component within this layer is the Proxy Server, which acts as an intermediary, facilitating secure and efficient communication between the fog nodes and the cloud server. The proxy server aggregates data from the fog nodes, ensuring streamlined data flow to the cloud.

At the top layer, the Cloud Layer, the cloud server provides centralized data processing, storage, and management. It performs advanced analytics, offers long-term data storage solutions, and implements comprehensive security measures. The architecture is designed to handle the entire data lifecycle, from collection by IoT devices to initial processing by fog nodes and advanced analysis by the cloud server.

By integrating these layers, the system provides a robust framework that supports efficient data processing, secure communication, and effective intrusion detection, thereby addressing the unique challenges posed by IoT-fog-cloud environments.

### Proposed anonymous authentication and intrusion dectection model

In the context of IoT and fog computing, ensuring secure and anonymous authentication while detecting intrusions is crucial. Traditional methods often fall short due to the limited computational resources of IoT devices and the need to preserve user privacy. To address these issues, we propose a novel model that combines anonymous authentication with a machine learning-based intrusion detection system. This model is designed to operate efficiently within the fog computing layer, providing robust security and maintaining user anonymity. Through a three-phase process—initial setup, user registration, and ongoing authentication—enhanced by advanced machine learning techniques, our model offers a scalable and effective solution for securing IoT-fog environments.

#### Proposed anonymous authentication method.

**Fig 2:**
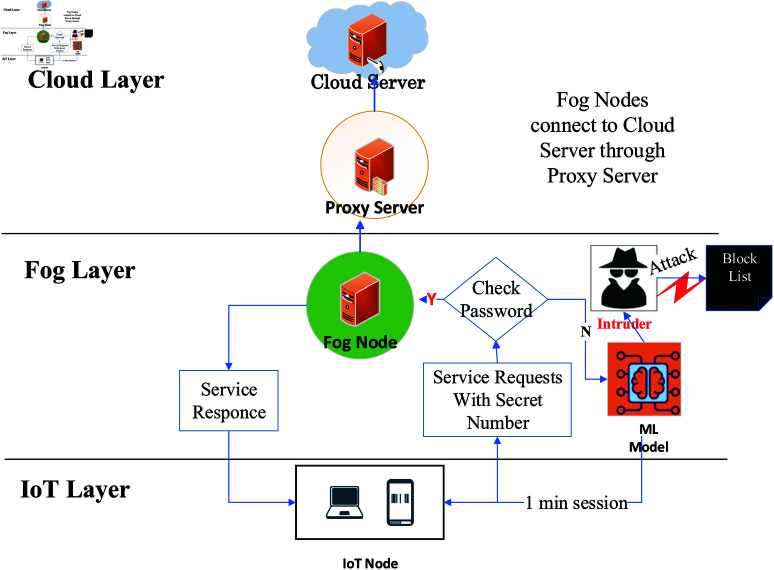
(a) Block diagram of anonymous authentication and intruder detection.

**Fig 3:**
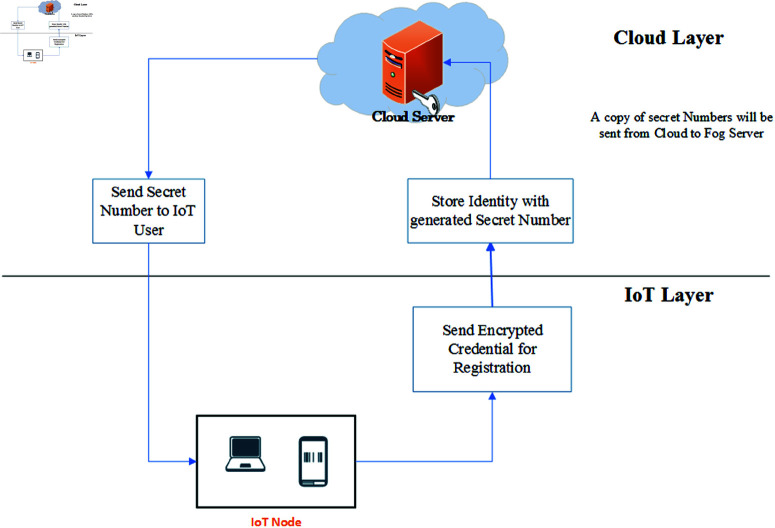
(b) Block diagram of anonymous authentication and intruder detection.

The proposed methodology involves three key components: cloud service providers (CSPs), fog service providers (FSPs), and IoT users. The system assumes the presence of multiple service providers and users within the fog-IoT ecosystem, acknowledging that some users may be malicious. Users and fog service providers are represented as $X=\{U_{i}|i=1,\ldots ,n\}$ and $Y=\{S_{j}|j=1,\ldots ,m\}$, respectively. The methodology is divided into three phases: initial, registration, and authentication. In the initial phase, private and public keys for service providers are generated to establish a secure communication framework. During the registration phase, IoT nodes or users register with a centralized cloud server, sharing their credential information securely. The cloud server generates a unique secret number for each user using the Cyclic Group of Prime Order [48], which is then distributed to the registered users and fog nodes, as depicted in Fig 2. Finally, in the authentication phase, users are authenticated by FSPs without sharing credential information, using a unique secret number. If a user inputs an incorrect secret number, they receive a one-minute service session during which a pre-trained machine learning model checks for intruders. Failure to provide the correct secret number results in the user being added to a block list, as shown in Fig 3. This methodology ensures secure, efficient management of IoT devices and services in a fog computing environment, addressing authentication and intrusion detection challenges.

#### Initial phase.

This part is essential for service providers and IoT users. It generates public and private key for the mentioned components. Steps of this phase are mentioned in Algorithm 1.

**Algorithm 1. Elliptic curve public and private key generation.**

1:Choose an elliptic curve E over a finite field ${𝔽}_{q}$ with a large prime order q.

2:Select a base point G on the curve E with order q.

3:Choose a random integer d (where 1≤d≤q−1) as the private key.

4:Compute the public key Q=d·G, where Q is a point on the curve E and · denotes point multiplication.

5:Publish $\left(E,{𝔽}_{q},G,Q\right)$ as the public key and retain d as the private key.

6:Send the public key to the requesters as needed.

#### Registration phase.

**Fig 4:**
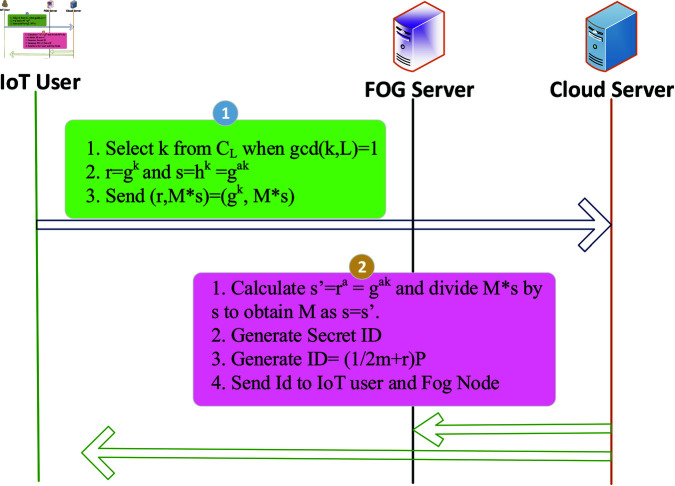
Registration phase.

The registration phase consists of two main steps: first, sending user credentials to the cloud service provider, and second, providing IoT users with a secret ID or number. The registration process for IoT users and the cloud service provider is illustrated in Fig 4.

**Sending credentials of users to cloud service provider:** The cloud service provider receives the encrypted credential information from the user for registration. The process is illustrated in Algorithm 2.

**Algorithm 2. Sending user credentials to cloud service provider with ECC.**

1:**Input:** Message M from user *U*_*i*_, Cloud Service Provider (CSP) with public key Q

2:User *U*_*i*_ selects a random integer k (where 1≤k≤q−1).

3:Compute R=k·G, where G is the base point on the elliptic curve.

4:Compute the shared secret S=k·Q, where Q is the CSP’s public key.

5:Encrypt the message M by computing $M^{\prime }=M\;⊕\;H(S)$, where H is a secure hash function and ⊕ is the XOR operation.

6:Send $\left(R,M^{\prime })=(k\;\cdot \;G,M\;⊕\;H(S)\right)$ and the public key Q to the CSP.

**Sending secret ID to IoT user and Fog Node:** The secret ID generated by the cloud service provider is sent to the IoT user. Algorithm 3 explains how an IoT user’s ID is sent by the cloud. It ensures that the secret ID is securely transmitted and can only be decrypted by authorized devices, thus maintaining security in the IoT-Fog-Cloud architecture.

**Algorithm 3. Sending secret ID to IoT user and fog node with ECC.**

1:**Step 1: Decrypting Message from IoT User**

2:CSP calculates the shared secret $S^{\prime }=d\;\cdot \;R$, where d is CSP’s private key and R is the received point from the IoT user.

3:CSP retrieves the original message M by calculating $M=M^{\prime }⊕H(S^{\prime })$, where H is a secure hash function.

4:**Step 2: Generating Secret ID**

5:Generate a random number r using a secure random generator.

6:Compute the secret ID using the formula $ID=\frac{P}{2m}\;+\;r$, where: P is a prime order, r is the random value and m is the unique user Id (prime number).

7:**Step 3: Secure Transmission of Secret ID**

8:Encrypt ID using ECC by computing $ID^{\prime }=ID\;⊕\;H(S)$, where $S=k\;\cdot \;Q_{IoT}$ and *Q*_*IoT*_ is the public key of the IoT user.

9:Send $\left(R_{ID},ID^{\prime })=(k\;\cdot \;G,ID\;⊕\;H(S)\right)$ to both the Fog Node and IoT user.

10:**Step 4: Decryption by IoT User and Fog Node**

11:IoT user and Fog Node calculate the shared secret $S=d_{IoT}\;\cdot \;R_{ID}$, where *d*_*IoT*_ is their respective private key.

12:Retrieve the secret ID by computing $ID=ID^{\prime }\;⊕\;H(S)$.

#### Authentication phase.

The authentication procedure is performed between the IoT user and fog service provider is shown in the Fig 2. If a IoT user *U*_*i*_ needs to communicate with the fog service provider *S*_*j*_, the anonymous authentication protocol is performed. The Algorithm 4 outlines a secure authentication protocol for IoT users using a challenge-response mechanism backed by Elliptic Curve Cryptography (ECC) and machine learning-based intrusion detection. Initially, the IoT user *U*_*i*_ initiates a service request by sending a hashed ID *H*(*ID*) to the service provider *S*_*j*_. In response, *S*_*j*_ generates a unique nonce *N*_*s*_ and timestamp *T*_*s*_, sending them back to *U*_*i*_ to set up a secure session. The user then verifies the timestamp’s validity and generates an ECC key pair $\left(d_{u},Q_{u}\right)$, computing a shared session key *K*_*session*_ with *S*_*j*_’s public key *Q*_*s*_. Using this session key, *U*_*i*_ encrypts the message *H*(*ID*) and the nonce *N*_*s*_, producing an encrypted message *E*, and sends both *Q*_*u*_ and *E* to *S*_*j*_.

**Algorithm 4. Enhanced IoT user authentication.**

1:**Step 1: Initial Request from User**

2:*U*_*i*_ initiates a service request by sending H(ID) to *S*_*j*_.

3:**Step 2: Challenge-Response Setup**

4:*S*_*j*_ generates a unique nonce *N*_*s*_ and a timestamp *T*_*s*_.

5:*S*_*j*_ sends the nonce *N*_*s*_ and timestamp *T*_*s*_ to *U*_*i*_ to initiate a secure session.

6:**Step 3: User Response**

7:*U*_*i*_ verifies *T*_*s*_ to ensure it’s valid.

8:*U*_*i*_ generates an ECC key pair $\left(d_{u},Q_{u}=d_{u}\cdot G\right)$ for the session.

9:Using the FSP’s public key *Q*_*s*_, *U*_*i*_ computes a shared session key $K_{session}=d_{u}\cdot Q_{s}$.

10:*U*_*i*_ encrypts H(ID) and *N*_*s*_ using *K*_*session*_, resulting in $E={\text{Enc}}_{K_{session}}(H(ID)||N_{s})$.

11:*U*_*i*_ sends (*Q*_*u*_,*E*) to FSP, where *Q*_*u*_ is the public key of *U*_*i*_ for this session.

12:**Step 4: Verification by Service Provider**

13:*S*_*j*_ checks the received *N*_*s*_ against its generated value to confirm the request is for the current session.

14:*S*_*j*_ decrypts and verifies the received E using H(ID) stored in DefIdStore.

15:If (successful verification):

16:*U*_*i*_ is authenticated and a secure session is established.

17:Else

18:*U*_*i*_ fails the authentication check.

19:Apply the Ensemble approach 5 to detect potential intruders.

20:If ML predicts as an intruder:

21:*U*_*i*_ is identified as an intruder and added to the block list.

22:EndIf

23:EndIf

Upon receiving this response, *S*_*j*_ checks *N*_*s*_ against the original nonce for session validity and decrypts *E* using *H*(*ID*) stored in its secure database. If *S*_*j*_ successfully verifies the session, *U*_*i*_ is authenticated, establishing a secure session. However, if the verification fails, an ensemble machine learning model is triggered to determine if the request is likely from an intruder. If the model detects suspicious behavior, *U*_*i*_ is classified as an intruder and added to a block list, preventing further access attempts. This combination of ECC, nonce-based session validation, and machine learning ensures robust security while mitigating replay and unauthorized access attacks.

Additionally, during the authentication process, an attacker may occasionally acquire the correct password or secret number, which could result in unauthorized access. To promptly identify such threats, employing an Intrusion Detection System (IDS) can effectively enhance security.

### Intrusion Detection System (IDS) in Fog-IoT environment

In this section, we introduce our stacked ensemble learning-based intrusion detection model for Fog-IoT environments. The process involves data preprocessing, including filling missing values and normalization, followed by feature selection and data partitioning into training and testing sets. The training data undergoes k-fold cross-validation. The model stack consists of Random Forest with grid search cross-validation, XGBoost, and AdaBoost, whose predictions are combined using Logistic Regression as the meta-learner. This comprehensive approach ensures accurate intrusion detection by leveraging multiple machine learning techniques.

#### Data preprocessing.

Data preprocessing is a crucial step in preparing the dataset for machine learning models, especially in the context of intrusion detection in Fog-IoT environments. The raw data collected from IoT devices often contain noise, missing values, and inconsistent formats, which can negatively impact the performance of machine learning algorithms. Therefore, a series of preprocessing steps are applied to ensure that the data is clean, consistent, and ready for analysis.

1. **Handling Missing Values:** The first step in data preprocessing involves identifying and addressing missing values in the dataset. Missing data can occur due to sensor errors, communication issues, or other unforeseen factors. Techniques such as mean/mode/median imputation have been employed to fill in the missing values. In some cases, rows or columns with a high proportion of missing data may be removed entirely to prevent bias in the model.
2. **Data Normalization:** After handling missing values, the next step is data normalization. IoT data can have features with varying scales, which may cause machine learning models to perform suboptimally. Normalization involves scaling the features to a standard range, typically between 0 and 1, or standardizing them to have a mean of 0 and a standard deviation of 1. It can be defined as (1):
  $$\rho_{new}=\frac{\rho -min(\rho )}{max(\rho )-min(\rho )}$$ (1)
  where, $\rho_{new}$=normalized data, ρ=old data, max(ρ)= minimum value of old data, minâ#129;¡(ρ)=maximum value of old data. This process ensures that all features contribute equally to the model’s learning process, improving convergence speed and accuracy.
3. **Feature Selection:** Not all features in the dataset may be relevant for intrusion detection. Feature selection aims to identify and retain only the most significant features that contribute to the model’s predictive power. Techniques such as correlation analysis, mutual information, and feature importance from the Chi-Squared test have been applied to select the optimal set of features. Reducing the dimensionality of the data in this way helps to improve model performance and reduce computational costs.
4. **Data Partitioning:** Once the data is cleaned and the relevant features are selected, it is partitioned into training and testing sets. Typically, 80% of the data is allocated for training the model, while the remaining 20% is used for testing its performance. This partitioning ensures that the model can be evaluated on unseen data, providing an accurate measure of its generalization ability.

By carefully preprocessing the data, we ensure that the machine learning models are trained on high-quality input, leading to more accurate and reliable intrusion detection in Fog-IoT environments.

#### Proposed stacked ensemble model.

**Fig 5:**
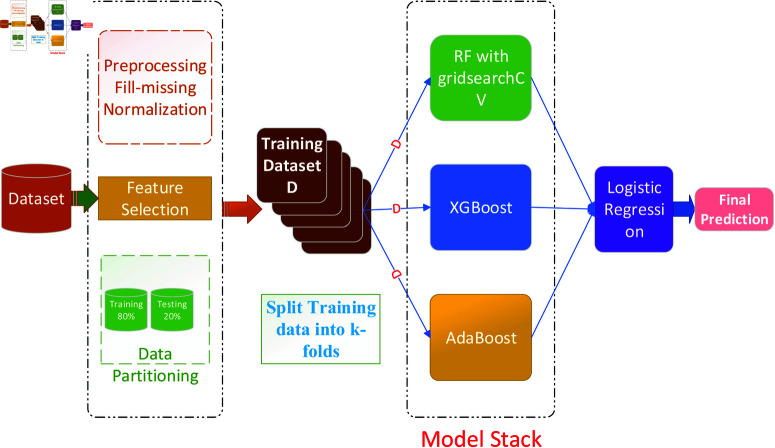
Architecture of proposed stacked ensemble learning model.

Stacked ensemble learning is an advanced machine learning technique that combines multiple different models (called base models) to improve predictive accuracy. These base models make predictions on the data, and a meta-learner then aggregates these predictions to produce a final output. This method leverages the strengths of diverse models, leading to improved accuracy, flexibility, and robustness compared to using a single model. Stacked ensembles are particularly useful in complex tasks where high accuracy is crucial. To optimize the performance of our stacked ensemble model [49], we first evaluate various baseline models and select the top three performers for inclusion at the base level (level 0) of the ensemble. The stacked ensemble is engineered to surpass the predictive accuracy of individual machine learning models. In our approach, we employ Logistic Regression (LR) as the super learner or meta-learner. The k-fold stacking process, depicted in Fig 5, begins by dividing the training dataset into k segments. Out of these, N-1 segments are used to train the individual classifiers, while one segment is reserved for testing. The base classifiers selected for this process include GridSearchCV with Random Forest, XGBoost (XGB), and AdaBoost classifier among Random Forest, GridSearchCV with Random Forest, XGBoost (XGB), Support Vector Classifier (SVC), Stochastic Gradient Descent (SGD), AdaBoost, and Gradient Boosting Machine (GBM).To determine the optimal ensemble from this collection of classifiers, we feed the test data of all base classifiers into a Logistic Regression (LR). Here is a brief description of the three models:

1. Random Forest with GridSearchCV: Random Forest (RF) is an ensemble learning method that operates by constructing a multitude of decision trees during training and outputting the mode of the classes (classification) or mean prediction (regression) of the individual trees. GridSearchCV is employed with RF to systematically search for the optimal hyperparameters, enhancing the model’s performance by selecting the most effective combination of parameters through cross-validation.
2. XGBoost: Extreme Gradient Boosting (XGBoost) is a powerful ensemble technique based on decision trees, designed for speed and performance. It implements gradient boosting algorithms in an efficient, scalable way, using a combination of model tuning and robust regularization to prevent overfitting. XGBoost is known for its accuracy and efficiency in handling large-scale datasets and complex data relationships.
3. AdaBoost: Adaptive Boosting (AdaBoost) is an ensemble method that combines multiple weak classifiers to create a strong classifier. It works by iteratively adjusting the weights of misclassified instances, focusing more on difficult cases in subsequent rounds. Each classifier is trained on the weighted dataset, and the final prediction is a weighted sum of the individual classifiers, improving the overall accuracy and robustness of the model.

Then, we can mathematically define our proposed model. The pair (*a*,*b*)_*k*_ of *k*-folds with $a=(a_{1},a_{2},....,a_{r})$ representing the *r* recorded values and $b=(b_{r+1},b_{r+2},.....,b_{r+p})$ defining the *p* values for prediction involves considering a set of *N* potential learning algorithms denoted as *model*_*i*_, i=1,.......,N. Consider *M*_*ij*_ as the model generated by the learning algorithm *model*_*i*_ on *a* to predict *a*_*p* + *j*_, and let ${\hat{y}}_{j}$ be the generalizer function responsible for combining the models to make predictions for such a value. Then, the probable ${\hat{a}}_{\left(p+j\right)}$ value can be defined by the Eq 2:

$${\hat{a}}_{\left(p+j\right)}={\hat{y}}_{j}(M_{ij},\ldots \ldots ,M_{Nj})$$ (2)

The logistic regression model for the meta-learner can be represented mathematically as (3):

$$\hat{y}=\sigma \left(\beta_{0}+\sum_{j=1}^{n}\beta_{j}M_{j}\right)$$ (3)

where: $\hat{y}$ is the final prediction from the meta-learner. σ(x) is the sigmoid function, defined as (4):

$$\sigma (x)=\frac{1}{1+e^{-x}}.$$ (4)

$\beta_{0}$ is the intercept term of the logistic regression model. $\beta_{j}$ are the coefficients learned by the logistic regression model for each base model’s prediction. *M*_*j*_ is the prediction made by the *j*-th base model in the ensemble and *n* is the number of base models in the ensemble.

### System performance metrics

The performance of our proposed authentication technique will be analyzed in terms of computational cost, latency, and network usage. Moreover, the intruder detection system will be evaluated in terms of accuracy, precision, recall, f1-score and ROC curve.

1. **Computation Cost:** The computation cost is computed as the time taken to authenticate one user or several users. The computation cost of the proposed scheme is defined as (5):
  $$C=2T_{h}+T_{d}+T_{p}+T_{ed}$$ (5)
  where, *T*_*h*_ =  time taken for performing a hash function, *T*_*p*_ =  time taken to perform pairing, *T*_*d*_ =  time taken to detect the user type, *T*_*ed*_ =  time taken to perform encryption/decryption.
2. **Latency:** In the context of user authentication, latency refers to the delay between when a user submits their credentials (such as a password, biometric scan, or other authentication factors) and when the system verifies these credentials and grants or denies access. Thus, latency can be written as (6):
  tial=C+η+τ (6)
  where *C* is the Tuple CPU Execution Delay for authentication and η is the time to upload credential on fog node for processing. Finally, τ is the time taken to response to the IoT user after processing at the Fog node.
3. **Communication Overhead:** The communication overhead in an IoT-Fog system can be expressed as the total amount of data exchanged between the IoT devices, Fog nodes, and the Cloud during the authentication process. This includes both the transmission of encrypted credentials (e.g., secret IDs) and the response from the server. The total communication overhead *O*_*C*_ is given by:
  $$O_{C}=n\cdot (D_{t}+D_{r})+D_{f}$$ (7)
  Where: *O*_*C*_ =  Total communication overhead (in bytes or KB), *n* =  Number of IoT devices, *D*_*t*_ =  Data size for each transmission from an IoT device to the Fog node (in bytes or KB), *D*_*r*_ =  Data size for each response from the Fog node to the IoT device (in bytes or KB), *D*_*f*_ =  Data size exchanged between the Fog node and the Cloud server (if needed) during the authentication process (in bytes or KB).
4. **Accuracy:** The proportion of true results (both true positives and true negatives) among the total number of cases examined. It measures the overall correctness of the model.
  $$Accuracy=\frac{TP+TN}{TP+TN+FP+FN}$$ (8)
5. **Precision:** The proportion of true positive results out of all the positive results predicted by the model. It indicates how many of the positive predictions were actually correct.
  $$Precision=\frac{TP}{TP+FP}$$ (9)
6. **Recall:** The proportion of true positive results out of all the actual positives. It shows how well the model can identify all relevant cases.
  $$Recall=\frac{TP}{TP+FN}$$ (10)
7. **F1-Score:** The harmonic mean of precision and recall. It provides a balanced measure, especially when there’s an uneven class distribution or when one wants to consider both precision and recall equally.
  $$F1-Score=\frac{2*Precision*Recall}{Precision+Recall}$$ (11)

## Experimental results and discussion

This section presents the experimental evaluation of the proposed anonymity-preserving scheme for IoT user authentication and the machine learning-based intrusion detection system in a Fog computing environment. We assess the performance of our model using various metrics, including accuracy, precision, recall, F1-score, and ROC analysis. Comparative analyses with traditional cloud-based schemes are also provided to highlight the efficiency and effectiveness of our approach. The results are thoroughly discussed to demonstrate the robustness and applicability of the proposed solution in real-world scenarios.

### Experimental setup

In this subsection, we detail the experimental setup used to evaluate the proposed anonymity-preserving authentication and machine learning-based intrusion detection model in a fog computing environment. The simulations were conducted using the iFogSim toolkit [50], a specialized tool for simulating and modeling fog computing environments.

The iFogSim environment was configured to simulate a multi-layer fog computing architecture, which includes IoT devices, fog nodes, and cloud servers. The simulation scenario is designed to mimic real-world fog-IoT networks, where IoT devices generate data that is processed by fog nodes, with cloud servers providing additional computational power and storage.

**Fig 6:**
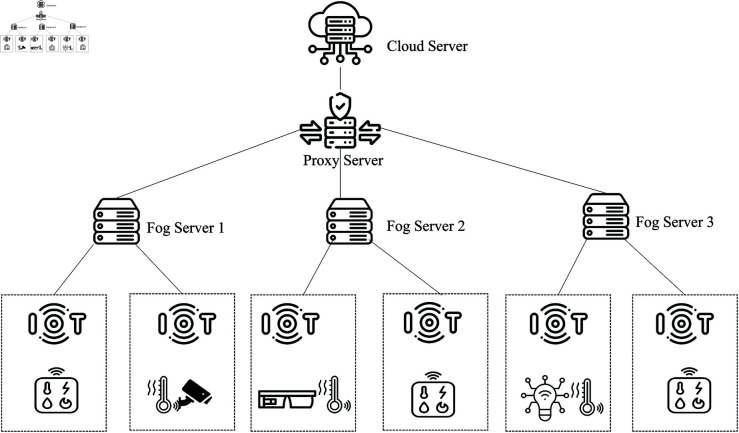
Initial iFogSim topology evaluation for the proposed approach.

Fig 6 illustrates the topology designed for evaluating the results in a fog computing scenario. The topology consists of three fog nodes, with each fog node connected to two IoT nodes. Furthermore, each IoT node is equipped with two sensors. This setup was specifically created to assess latency and computation costs within the iFogSim environment, changing the number of IoT nodes.

Table 1 represents the configuration parameters of the fog server, proxy server, and cloud server established during the fog-based scenario simulation. The setup settings encompass the following: delay, busy power and idle power, Random Access Memory (RAM), uplink and downlink bandwidth, level, rate, or cost of processing one million instructions, and processing capabilities in terms of instructions per million.

**Table 1:** Value of parameters of IoT, Fog, Proxy, and Cloud node for simulation.

Description	Parameter	Value
Cloud		
Upstream Bandwidth	*U*_*B*_(MB)	10000 MB
Downstream Bandwidth	*D*_*B*_(MB)	10000 MB
Storage	RAM(MB)	32000 MB
Processing Power	CPU(MIPS)	34000 MIPS
Level	Tier	0
Latency to Proxy Server	$L_{C\sim Ps}$	100 ms
Proxy Server		
Upstream Bandwidth	*U*_*B*_(MB)	2000 MB
Downstream Bandwidth	*D*_*B*_(MB)	2000 MB
Storage	RAM(MB)	8000 MB
Processing Power	CPU(MIPS)	5000 MIPS
Level	Tier	1
Fog Node		
Upstream Bandwidth	*U*_*B*_(MB)	1000 MB
Downstream Bandwidth	*D*_*B*_(MB)	1000 MB
Storage	RAM(MB)	4000 MB
Processing Power	CPU(MIPS)	2800 MIPS
Latency to Proxy Server	$L_{F\sim Ps}$	4 ms
Latency to IoT Node	$L_{F\sim In}$	1 ms
Level	Tier	2
Busy Power	*B*_*p*_(W)	300 Watt
Idle Power	*I*_*p*_(W)	75 Watt
IoT Node		
Upstream Bandwidth	*U*_*B*_(MB)	200 MB
Downstream Bandwidth	*D*_*B*_(MB)	250 MB
Storage	RAM(MB)	2048 MB
Processing Power	CPU(MIPS)	1500 MIPS
Level	Tier	3
Busy Power	*B*_*p*_(W)	245 Watt
Idle Power	*I*_*p*_(W)	65 Watt

To integrate a pretrained machine learning (ML) model with the iFogSim simulator, we must first ensure the model is in a Java-compatible format, such as a serialized Java object, and include any necessary dependencies within the iFogSim project. The next step involves setting up the environment by installing required Java ML libraries and modifying relevant iFogSim classes, particularly those related to fog nodes, to accommodate the ML model. Once the environment is prepared, we can write code to load the pretrained model into the iFogSim framework. With the model loaded, we can integrate it with fog nodes, enabling these nodes to preprocess incoming data and pass it through the ML model for predictions. The fog nodes’ behavior can then be adjusted based on these predictions, simulating real-time decision-making processes. After integration, we can run simulations within iFogSim to evaluate the ML model’s impact on key performance metrics like latency and accuracy. Based on these simulation results, further optimization of the ML model and simulation settings can be carried out to enhance overall system performance in the Fog IoT environment.

### Evaluation of authentication scheme

**Fig 7:**
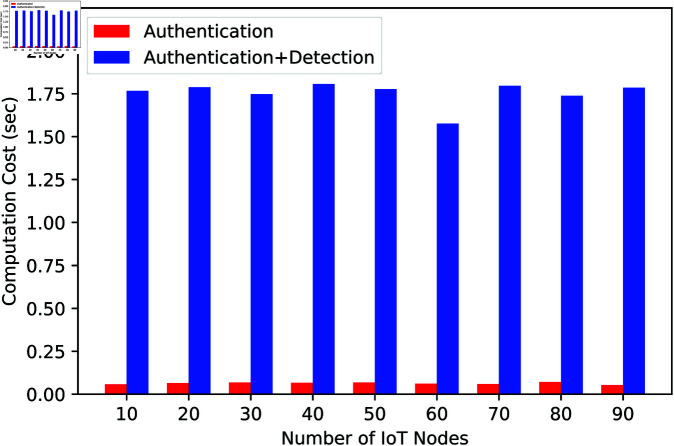
Computational cost of the proposed approach.

The anonymous authentication scheme has been evaluated in different metrics, such as computational cost, latency, throughput, etc. Fig 7 presents the computation cost for user authentication in a Fog-IoT environment, comparing scenarios with and without intrusion detection. The x-axis shows the number of IoT nodes, and the y-axis indicates the computation cost in seconds. The computation cost without intrusion detection remains consistently low, between 0.0057 and 0.0071 seconds, regardless of the number of IoT nodes, highlighting the efficiency of the basic authentication process. In contrast, when intrusion detection is included, the computation cost increases significantly, ranging from approximately 1.5761 to 1.8066 seconds. Despite this increase, the cost remains stable as the number of IoT nodes grows, indicating that the system’s performance does not degrade significantly with additional nodes. This suggests that while intrusion detection adds overhead, the system handles it effectively without a major impact on scalability.

**Fig 8:**
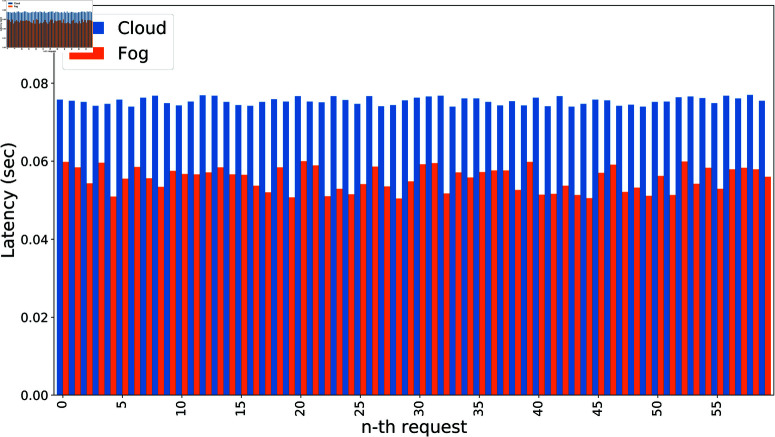
Comparison of latency for the authentication process.

Latency refers to the delay in network communication. Fig 8 presents a comparison of network delays between the fog and cloud schemes. The graph illustrates that the latency in the fog scheme is consistently lower than in the cloud scheme. The x-axis represents the number of n-th requests, while the y-axis shows the average network delay for each request. The cloud scheme’s delays range between 0.074 and 0.077 seconds, whereas the fog scheme achieves lower delays, ranging from 0.045 to 0.054 seconds, demonstrating a significant reduction in latency.

**Fig 9:**
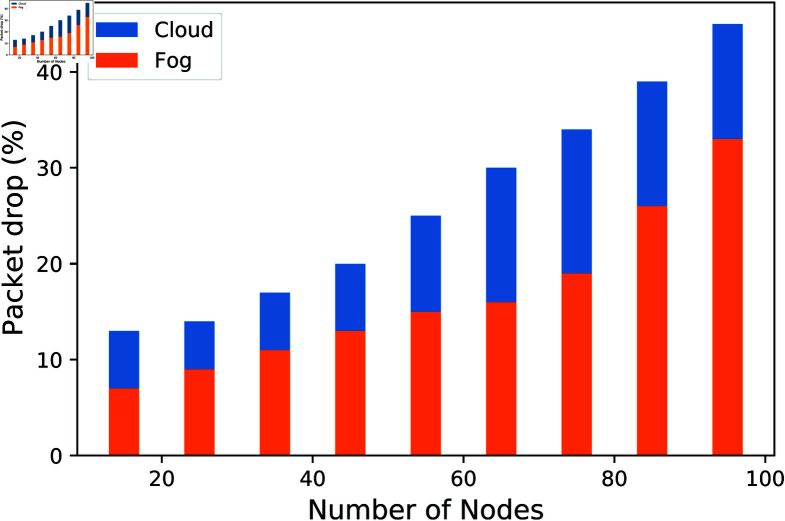
Comparison of packet drop in the authentication process.

The text describes how the packet drop rate, expressed as a percentage of sent packets, is compared between the fog and cloud schemes. Fig 9 demonstrates that the packet drop ratio in the fog scheme is generally lower than in the cloud scheme. However, when the number of nodes exceeds 90, the packet drop percentages of both schemes become nearly identical. This indicates that the fog scheme outperforms the cloud scheme in terms of packet drop ratio, particularly when the number of nodes is fewer than 90.

**Fig 10:**
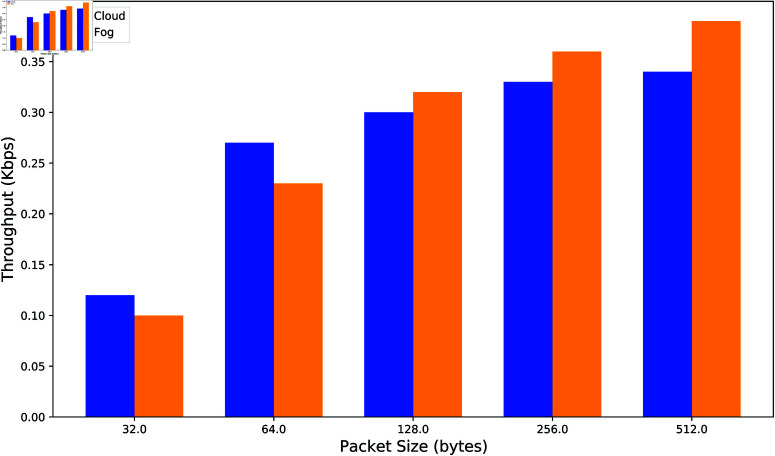
Comparison of throughput in the authentication process.

An increase in throughput generally indicates a more effective scheme. The x-axis represents packet size in bytes, while the y-axis shows throughput in kbps. The throughput of the fog scheme is compared with that of the cloud scheme, as depicted in Fig 10. The results indicate that the fog scheme generally achieves better throughput than the cloud scheme. Although the cloud scheme outperforms the fog scheme when the packet size is less than 128 bytes, the fog scheme demonstrates superior throughput for packet sizes greater than 128 bytes.

**Fig 11:**
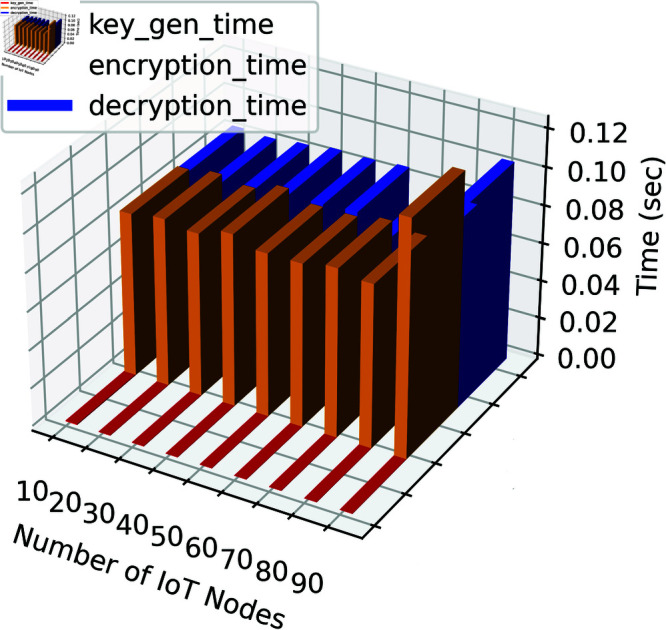
Performance of the proposed authentication algorithm.

Fig 11 shows the performance of the proposed authentication algorithm for different numbers of IoT nodes, specifically focusing on the times taken for key generation, encryption, and decryption processes. Each set of bars represents a unique IoT node count, ranging from 10 to 90, with key generation, encryption, and decryption times shown in blue, green, and red, respectively. Key generation times remain consistently low across all node counts, with values such as 0.000400 seconds for 10 nodes and decreasing slightly to 0.000150 seconds for 20 and higher node counts, showcasing the efficiency of ECC in generating keys. Encryption times vary slightly with node count, starting at 0.085660 seconds for 10 nodes and peaking at 0.123494 seconds for 90 nodes. Decryption times follow a similar trend, ranging from 0.084760 seconds for 10 nodes to 0.104679 seconds for 90 nodes. These values indicate that, while encryption and decryption processes scale effectively, higher workloads introduce modest performance trade-offs. Overall, the figure highlights that the proposed authentication scheme maintains low computational overhead even as the IoT network grows.

**Fig 12:**
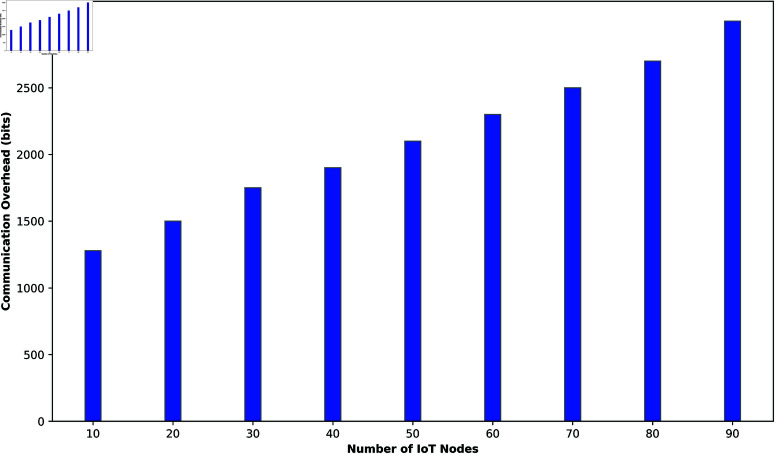
Communication overhead of the proposed authentication framework.

Fig 12 illustrates the communication overhead of the proposed authentication framework as the number of IoT nodes increases from 10 to 90. Each bar represents the communication cost, measured in bits, for a given IoT node count. As shown, the communication overhead starts at 1280 bits for 10 IoT nodes and gradually increases with node count, reaching 1500 bits at 20 nodes and 1750 bits at 30 nodes. This trend continues, with the overhead rising to 1900 bits at 40 nodes, 2100 bits at 50 nodes, and 2300 bits at 60 nodes. At higher node counts, the communication cost grows to 2500 bits for 70 nodes, 2700 bits for 80 nodes, and peaks at 3000 bits for 90 nodes. This increase in communication overhead reflects the additional data exchanged during key generation, challenge-response, encryption, and verification phases as more devices join the network. The figure highlights the scalability of the authentication framework, as it maintains relatively low overhead per node even as the number of IoT devices expands significantly. The overhead remains within acceptable limits, demonstrating the efficiency of the framework in large-scale IoT environments.

### Security analysis of the proposed authentication model

To provide a comprehensive Security Analysis of the proposed authentication model, we use the Real-or-Random (RoR) security model and Canetti-Krawczyk (CK) Model to evaluate its effectiveness against various types of attacks. The RoR model is widely used to validate the indistinguishability of a protocol session from a random session, which helps prove that an adversary cannot derive meaningful information, even if they have access to intercepted messages. On the other hand, the CK Model is a widely accepted framework for analyzing the security of key exchange and authentication protocols.

In the RoR model, a challenger interacts with an adversary, presenting it with either the real execution of the protocol (Real world) or a randomly simulated execution (Random world). The adversary’s goal is to distinguish between these two worlds. If the adversary cannot reliably distinguish between the real and random sessions, the protocol is deemed secure. The proposed authentication protocol ensures robust security by addressing key vulnerabilities using advanced mechanisms. It employs Elliptic Curve Cryptography (ECC) to encrypt communications between IoT devices, Fog nodes, and the Cloud, ensuring that intercepted data cannot be decrypted without the private key, thereby maintaining confidentiality. A nonce and timestamp-based challenge-response mechanism prevents replay attacks by ensuring session freshness, while ECC-derived session keys safeguard against Man-in-the-Middle (MITM) attacks by preventing unauthorized decryption or message tampering. Additionally, the protocol integrates a machine learning model, described in the next section, trained on the IoTID20 dataset to detect adversarial behaviors, such as Mirai and Brute Force attacks, providing an additional layer of defense by flagging and blocking suspicious activities. Importantly, encryption keys are never transmitted directly, and session keys are uniquely derived for each session using ECC, ensuring that private keys remain secure and the system is resistant to tampering or key exposure. This comprehensive approach effectively protects the protocol against a wide range of attacks.

**Fig 13:**
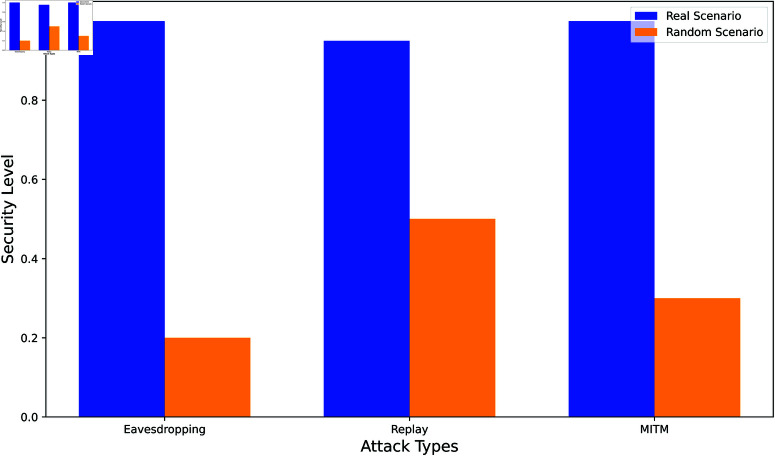
Real vs. random scenario analysis for different attacks.

Fig 13 illustrates the comparative security levels of three types of attacks—Eavesdropping, Replay, and Man-in-the-Middle (MITM)—under Real and Random scenarios within the proposed authentication framework. The Real scenario, represented by blue bars, consistently shows higher security values across all attack types, with values close to 1, indicating effective protection mechanisms. This high level of security demonstrates the robustness of the protocol in authenticating users and securing communication against potential attacks. In contrast, the Random scenario, represented by orange-colored bars, shows significantly lower security levels, with values ranging from 0.2 to 0.5. These lower values indicate that, without the security controls in place, such as ECC-based encryption and nonce usage, the protocol is more vulnerable to these types of attacks. This comparison underscores the effectiveness of the proposed model, particularly in protecting against eavesdropping and MITM attacks, by maintaining high security under Real conditions.

It ensures secure communication in the presence of adversaries with capabilities such as eavesdropping, message tampering, replay attacks, and session hijacking. Here, we analyze the proposed authentication model using the CK Model to validate its security guarantees. The proposed authentication model satisfies key security properties essential for robust protection in IoT and Fog environments. Session Key Security is achieved through ECC-based key derivation, ensuring session keys are indistinguishable from random and never transmitted, safeguarding them from adversaries. Mutual Authentication is established using a nonce-based challenge-response mechanism and validation of the user’s hashed ID, ensuring both parties verify each other. Replay Attack Resistance is ensured by employing nonces (*N*_*s*_) and timestamps (*T*_*s*_), which guarantee session freshness and prevent the reuse of intercepted messages. The model also provides Forward Secrecy, ensuring past session keys remain secure even if private keys are compromised, through the use of session-specific ECC key generation and ephemeral public keys. Additionally, MITM Attack Resistance is achieved via ECC-derived session keys and encrypted message exchanges, which protect the integrity and confidentiality of communication, preventing unauthorized modifications or forgery. Together, these properties make the proposed model a robust solution for secure authentication in IoT systems.

**Fig 14:**
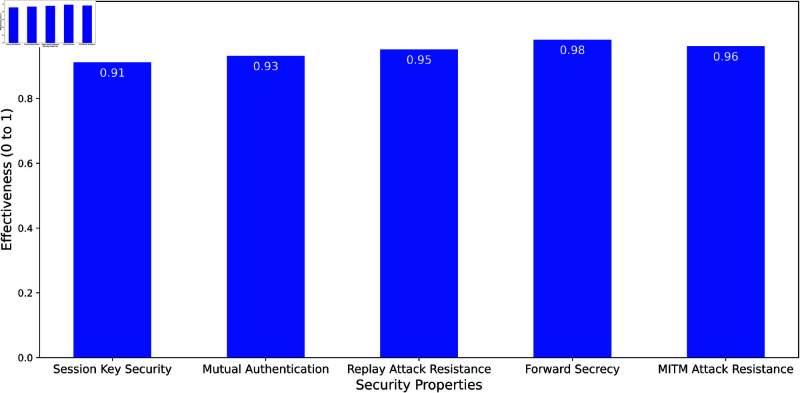
CK model analysis of proposed authentication framework.

The bar Fig 14 demonstrates the effectiveness of the proposed authentication model across key security properties as evaluated using the CK (Canetti-Krawczyk) Model, with effectiveness scores ranging from 0.91 to 0.98. Session Key Security achieves a score of 0.91, indicating strong protection of session keys, ensuring they are indistinguishable from random values and secure against adversaries. Mutual Authentication scores 0.93, highlighting the model’s reliability in verifying the identities of both IoT users and service providers. Replay Attack Resistance, with a score of 0.95, reflects the efficacy of the nonce and timestamp mechanism in preventing the reuse of intercepted messages. The highest score, 0.98, is achieved for Forward Secrecy, underscoring the protocol’s ability to protect past session keys even if private keys are compromised. Lastly, MITM Attack Resistance scores 0.96, showcasing the model’s robust defense against man-in-the-middle attacks by ensuring message integrity and confidentiality. Overall, the chart highlights the high effectiveness of the proposed model in addressing critical security concerns in IoT and Fog computing environments.

### Evaluation of intrusion detection system

The goal of this study is to detect intrusions in user activities by utilizing the IoTID20 [51] dataset and exploring various machine learning techniques. The research was conducted on a computing system powered by an 8th generation Intel Core i7 6600U processor, clocked at up to 3.1 GHz, with 16 GB of RAM. In this section, we thoroughly evaluate and compare the performance of our proposed stacked machine learning model for intrusion detection. The evaluation is conducted across multiple key dimensions, offering a detailed analysis of the model’s robustness and effectiveness.

#### Data preprocessing and feature selection.

The IoTID20 dataset [51], comprising 83 network features and 3 label features across 625,783 instances (with 40,073 normal and 585,710 anomaly cases), underwent rigorous data preprocessing and feature selection to prepare it for model training. The preprocessing phase involved data normalization to ensure uniformity in feature scaling, enhancing the model’s performance. Additionally, duplicate data entries were removed to prevent redundancy and reduce computational overhead. After applying the data preprocessing techniques, the size of the dataset was reduced to 279690. To optimize the model, it is essential to reduce dimensionality, which plays a critical role in minimizing the number of features. We employed a Chi-squared test and correlation analysis to select the 40 most significant features from the original 83. The selected features— F0(Flow_ID), F2(Src_Port), F4(Dst_Port), F5(Protocol), F6(Timestamp), F9(Tot_Bwd_Pkts), F11(TotLen_Bwd_Pkts), F12(Fwd_Pkt_Len_Max), F13(Fwd_Pkt_Len_Min), F14(Fwd_Pkt_Len_Mean), F15(Fwd_Pkt_Len_Std), F16(Bwd_Pkt_Len_Max), F17(Bwd_Pkt_Len_Min), F18(Bwd_Pkt_Len_Mean), F20(Flow_Bytss), F21(Flow_Pktss), F23(Flow_IAT_Std), F24(Flow_IAT_Max), F30(Fwd_IAT_Min), F31(Bwd_IAT_Tot), F33(Bwd_IAT_Std), F34(Bwd_IAT_Max), F41(Bwd_Header_Len), F42(Fwd_Pktss), F43(Bwd_Pktss), F44(Pkt_Len_Min), F45(Pkt_Len_Max), F46(Pkt_Len_Mean), F47(Pkt_Len_Std), F49(FIN_Flag_Cnt), F51(RST_Flag_Cnt), F52(PSH_Flag_Cnt), F5(DownUp_Ratio), F58(Pkt_Size_Avg), F59(Fwd_Seg_Size_Avg), F67(Subflow_Fwd_Pkts), F71(Init_Fwd_Win_Byts), F78(Active_Min), F80(Idle_Std), and F81(Idle_Max)— were identified as the most relevant for distinguishing between normal (1) and anomaly (0) network activities, considering the p-value less than 0.05 (*p*<0.01). A brief statistics of the dataset is presented in Table 2. These features formed the basis for training the proposed intrusion detection model, ensuring both efficiency and accuracy in the classification process.

**Table 2:** Instances of normal and attacked in the IoTID20 Dataset [51].

Binary	Subcategory	Instance
Normal	Normal	40073
Anomaly	DoS	59391
	Mirai Ack Flooding	55124
	Mirai Brute force	121181
	Mirai HTTP Flooding	55818
	Mirai UDP Flooding	183554
	MITM	35377
	Scan Host Port	22192
	Scan Port OS	53073

#### Hyperparameter tuning.

Hyperparameter tuning involves optimizing the preset parameters of a machine learning model, which are not learned during training. These hyperparameters, such as learning rate and model complexity, significantly impact the model’s performance and must be set before training begins. The hyperparameter tuning process for the XGBoost model involved optimizing several parameters. With 2000 estimators, the model iteratively improves through 2000 boosting rounds, using XGBClassifier as the base estimator. A learning rate of 0.01 was chosen to ensure gradual learning, minimizing overfitting, while the gamma parameter was set to 0.4 to control tree complexity.

For the Random Forest model, GridSearchCV was used to find the best combination of parameters, setting the number of estimators to 200. The max_features parameter was set to log2, balancing complexity and performance by considering a logarithmic number of features for each split.

In the AdaBoost model, 50 estimators were used, combining 50 weak learners with a Random Forest as the base estimator. A learning rate of 1 was applied, controlling the influence of each weak learner on the final model. In all three base models, hyperparameter tuning was critical in achieving an optimal balance between bias and variance, enhancing the model’s ability to generalize to new data.

#### Performance evaluation of the proposed model.

In this part, we have evaluated the performance of our proposed staked ensemble model for intrusion detection using different techniques such as accuracy, f1-score, confusion matrix, ROC curve.

Performance comparison of different ML models.

**Fig 15:**
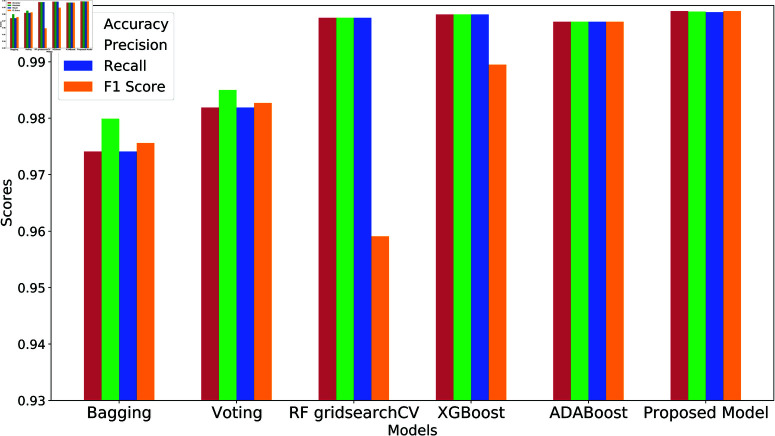
Performance comparison of different ML models.

Fig 15 compares the performance of various machine learning models for intrusion detection, including Bagging, Voting, RF GridSearchCV, XGBoost, AdaBoost, and the proposed model. Bagging achieves solid results with an accuracy of 0.9741 and an F1 score of 0.9756, but it is outperformed by Voting, which shows a slight improvement with an accuracy of 0.9819 and an F1 score of 0.9827. RF GridSearchCV delivers a high accuracy of 0.9978, though its F1 score drops to 0.9591, indicating some imbalance. XGBoost further improves performance, achieving an accuracy of 0.9984 and an F1 score of 0.9895, reflecting its strong classification capabilities. AdaBoost maintains consistent performance across all metrics, with an accuracy of 0.9971. However, the proposed model surpasses all others, demonstrating near-perfect performance with an accuracy of 0.9986 and an F1 score of 0.9990, indicating its superior effectiveness in detecting intrusions. The performance metrics of applied machine learning models are presented in Table 3 at a glance.

**Table 3:** Performance metrics of applied ML models.

Model	Accuracy	Precision	Recall	F1 Score
Bagging	0.9741	0.9799	0.9741	0.9756
Voting	0.9819	0.9850	0.9819	0.9827
RF gridsearchCV	0.9978	0.9978	0.9978	0.9591
XGBoost	0.9984	0.9984	0.9984	0.9895
ADABoost	0.9971	0.9971	0.9971	0.9971
Proposed Model	0.9986	0.9984	0.9985	0.9990

The classification report in Table 4 for the proposed model highlights its outstanding performance across various metrics. The model achieved a precision of 0.9989, a recall of 0.9996, and an F1-score of 0.9991 for detecting anomalous instances, with a support count of 50,376. For normal instances, the precision was 0.9977, recall was 0.9972, and the F1-score was 0.9949, with a support count of 5,540. The overall accuracy of the model was recorded at 0.9986, reflecting its exceptional reliability. The macro average, which accounts for both classes equally, yielded a precision of 0.9987, recall of 0.9944, and an F1-score of 0.9965. The weighted average, which considers the class imbalance, also resulted in high scores of 0.9984 precision, 0.9986 recall, and 0.9990 F1-score, underscoring the model’s robust ability to accurately classify both normal and anomalous data in the IoT environment.

**Table 4:** Classification Report for the proposed model.

Class	precision	recall	f1-score	Support
Anomaly	0.9989	0.9996	0.9991	50376
Normal	0.9977	0.9972	0.9949	5540
accuracy			0.9986	55916
macro avg	0.9987	0.9944	0.9965	55916
weighted avg	0.9984	0.9986	0.9990	55916

**Fig 16:**
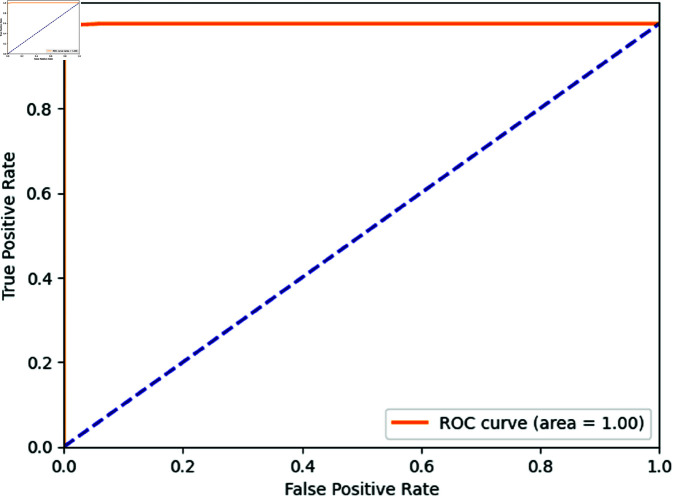
ROC curve of the proposed model.

The ROC (Receiver Operating Characteristic) curve for the proposed stacked ensemble model in Fig 16 demonstrates its strong performance in intrusion detection. The curve plots the True Positive Rate (TPR) against the False Positive Rate (FPR), with the diagonal blue dashed line representing a random classifier (where TPR equals FPR). The orange curve represents the performance of the proposed model, which closely hugs the top left corner, indicating that the model achieves a high true positive rate with a very low false positive rate. The area under the curve (AUC) is nearly 1.00, which suggests that the model has an excellent ability to distinguish between normal and anomalous instances, confirming its effectiveness for intrusion detection in IoT environments.

**Fig 17:**
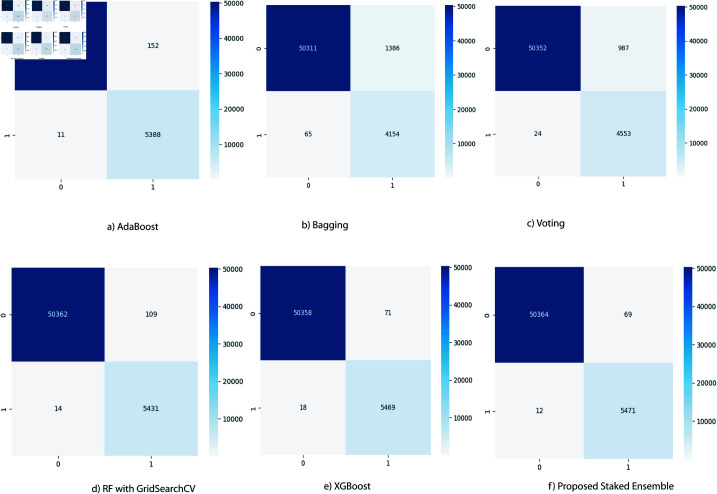
Confusion Matrix of different ML models.

The confusion matrix provides a comprehensive assessment of the model’s performance by detailing the number of true positives (TP), true negatives (TN), false positives (FP), and false negatives (FN), as illustrated in Fig 17. The AdaBoost classifier demonstrates strong predictive power with 50,365 true positives and 5,388 true negatives while recording only 11 false positives and 152 false negatives. The Bagging classifier also performs well, achieving 50,311 true positives and 4,154 true negatives, but with higher false positive (65) and false negative (1,186) counts. The RF with GridSearchCV and XGBoost classifiers further enhance performance, delivering 50,362 and 50,358 true positives, respectively, alongside 5,431 and 5,469 true negatives. Their false positive and false negative rates are notably low, with RF recording 14 false positives and 109 false negatives, and XGBoost 18 false positives and 71 false negatives. The Voting classifier achieves 50,352 true positives and 4,553 true negatives, with 24 false positives and 987 false negatives. The proposed stacked ensemble model excels, securing 50,364 true positives and 5,371 true negatives, with just 12 false positives and 69 false negatives. These results underscore the model’s superior ability to accurately distinguish between normal and anomalous instances.

Table 5 presents a comparative analysis of various anonymous authentication and intrusion detection models across different paradigms, focusing on key metrics such as user anonymity, computation cost, and detection accuracy. The table includes the latest research, showcasing a wide range of computation costs, from as low as 5.39 milliseconds in IoT-Cloud paradigms to 2.9 seconds in Fog-IoT systems. Detection accuracies vary among the models, with some achieving up to 99.86%.

**Table 5:** Comparison with existing work.

Ref.	Year	Anonymity	Cost	Paradigm	Detection Model	Accuracy
[13]	2023	Yes	5.94 ms	IoT	-	-
[30]	2024	Yes	5.39 ms	IoT-Cloud	-	-
[32]	2024	Yes	155 ms	Fog	-	-
[29]	2023	Yes	22.02 ms	Fog	-	-
[31]	2024	Yes		IoT	-	-
[28]	2024	Yes	14.22 ms	IoT	-	-
[38]	2023	No	-	Fog-to-Cloud	DNN-kNN	99.77%
[39]	2023	No	-	Fog-Cloud-IoT	RNN and BiLSTM	99.55%
[40]	2023	No	2.9 s	Fog-IoT	BiGAN	82.3%
[36]	2024	No	-	Fog-IoT	Transformer-CNN-LSTM	99%
[37]	2024	No	17 ms	Fog-IoT	DAE and BiLSTM	99.8%
Proposed Model	2024	Yes	1.75 s (Au+Det)	Fog-Cloud-IoT	Stacked Ensemble	99.86%

Note: In the cost column of our proposed model, (Au+Det) represents both authentication and intrusion detection time.

The proposed stacked ensemble model stands out by outperforming existing systems in several critical aspects. First and foremost, it achieves the highest detection accuracy at 99.86%, demonstrating its exceptional capability in accurately distinguishing between normal and anomalous activities within the network. Such high accuracy is vital for real-time intrusion detection, where the impact of false positives or false negatives can be severe.

Secondly, while the computation cost of the proposed model is 1.75 seconds—of which 5.3 milliseconds is dedicated to anonymous authentication, with the remaining time allocated to intrusion detection in the subsequent step for suspicious users—this cost remains within an acceptable range for many Fog-Cloud-IoT applications. The modest increase in computation time is justifiable due to the significant improvement in detection accuracy. This trade-off is essential to effectively handle the complexity and diversity of data in a Fog-Cloud-IoT environment.

In comparison, models employing simpler architectures, such as BiGAN [40], often achieve lower accuracy levels, which may not suffice for critical security applications. The proposed model’s balance between computation cost and accuracy renders it a robust solution for secure and efficient intrusion detection in a Fog-Cloud-IoT paradigm, particularly in scenarios where high accuracy is indispensable. Additionally, the model integrates a two-step authentication process—beginning with anonymous authentication using a secret ID. If a user fails to provide the correct ID, they are further assessed by the intrusion detection algorithm to determine whether they are an attacker. This integration of authentication with the ML model in the Fog-IoT paradigm enhances the system’s overall efficiency and security, positioning it as a more advanced and convenient solution compared to existing systems.

## Discussion and future direction

The proposed anonymity-preserving scheme for IoT users’ authentication and machine learning-based intrusion detection in Fog Computing has demonstrated significant improvements in both security and efficiency. By integrating anonymous authentication with a stacked ensemble machine learning model developed with three base models: RF with GridSearchCV, XGBoost, and AdaBoost while LR has been used as meta learner, the system effectively reduces unauthorized access and enhances the detection of anomalous activities. Encryption and hashing techniques have been employed to communicate credentials. The two-step authentication process, which combines secret number verification with intrusion detection, further strengthens security measures, making it a robust solution for Fog-Cloud-IoT environments. Users need to submit the secret number or ID, previously given from the Cloud server at the time of registration, to the Fog node. In this case, if a user fails to submit the correct secret ID, he/she will get a session to resubmit. In some sessions, an attacker might manage to obtain the correct ID or secret number, potentially leading to unauthorized access. However, by verifying the request with an Intrusion Detection System (IDS), the security level can be significantly enhanced and only normal users would be able to get access to the Fog servers. By meticulous analysis in the result section, we have shown that our proposed model outperformed with 99.86% accuracy and other performance metrics like throughput, latency, execution cost, etc. were also better. Additionally, it is capable of managing complexity generated in the Fog-Cloud-IoT environment by leveraging the strengths of different algorithms to capture various patterns in the data.

However, there are areas that demand further exploration. For instance, while the current model shows high accuracy and acceptable computation costs, optimizing the computation time without compromising security remains an ongoing challenge. Future research could focus on reducing the computational overhead associated with intrusion detection, possibly through more advanced feature selection techniques or by incorporating lightweight machine learning algorithms.

Additionally, the integration of privacy-preserving techniques, such as homomorphic encryption or federated learning, could be explored to enhance data security during the authentication and intrusion detection processes. As IoT networks continue to expand, ensuring scalability and adaptability of the proposed system will be crucial. Future work could also investigate the application of the proposed scheme in real-world scenarios, where the diversity of IoT devices and varying network conditions may present new challenges.

Our proposed authentication scheme is a robust solution tailored for the unique security and efficiency needs of IoT and fog computing environments. By employing a dual approach of ECC-based encryption and a machine learning-backed intrusion detection mechanism, it addresses critical challenges in privacy, scalability, and resource constraints. The use of ECC minimizes computational and communication overhead, making it ideal for IoT devices with limited processing power, while the machine learning component adds an adaptive layer of security by identifying potential intrusions in real-time. The inclusion of nonce and timestamp-based challenge-response techniques further enhances resilience against replay attacks. Overall, this scheme strikes a careful balance between maintaining low communication costs and achieving high levels of security, ensuring a scalable and efficient solution for large-scale IoT networks.

## Conclusions

In this research, we introduce an anonymity-preserving scheme for IoT users based on an authentication protocol and intrusion detection system in a Fog computing environment. Our proposed authentication scheme offers a robust solution designed specifically to meet distinct security and efficiency requirements. It utilizes a dual approach, combining ECC-based encryption with a machine learning-powered intrusion detection system. The experimental results, obtained using the iFogSim simulation tool, show that our proposed model surpasses traditional cloud-based approaches in terms of latency, packet drop ratio, and throughput while making a more convenient solution for real-time IoT applications. The model’s high accuracy (99.86%), precision (99.84%), recall (99.86%), and F1-score (99.9%) further affirm its robustness and capability to operate effectively under anonymity conditions. Additionally, the ROC analysis indicates an outstanding discriminatory power of the model in identifying both normal and anomalous traffic.

Finally, our proposed model defines a stacked ensemble machine learning-based intrusion detection and authentication scheme for preserving IoT user anonymity. The developed model helps to provide enhance security and privacy on the basis of high anonymity detection accuracy for the IoT users in Fog computing environments.
